# Worldwide Index of Serotype-Specific Pneumococcal Antibody Responses (WISSPAR): A curated database of clinical trial data

**DOI:** 10.12688/gatesopenres.14768.1

**Published:** 2023-07-13

**Authors:** Stephanie Perniciaro, Dominic Cooper-Wooton, Maria Knoll, Daniel Weinberger

**Affiliations:** 1 Epidemiology of Microbial Diseases, Yale University, New Haven, Connecticut, USA; 2 International Vaccine Access Center, Department of International Health, Johns Hopkins University, Baltimore, Maryland, USA

**Keywords:** clinical trials, pneumococcal disease, pneumococcal conjugate vaccines, immunogenicity, Streptococcus pneumoniae

## Abstract

The Worldwide Index of Serotype Specific Pneumococcal Antibody Responses (WISSPAR; https://wisspar.com), is a centralized, online platform housing data on immunogenicity from clinical trials of pneumococcal vaccines. The data on WISSPAR are primarily curated from outcomes tables from clinical trials and are made available in a searchable format that can be readily used for downstream analyses. The WISSPAR database includes trials covering numerous vaccine products, manufacturers, dosing schedules, age groups, immunocompromised groups, and geographic regions. Customizable data visualization tools are embedded within the site, or the data can be exported for further analyses. Users can also browse summary information about the clinical trials and their results. WISSPAR provides a platform for analysts and policy makers to efficiently gather, compare, and collate clinical trial data about pneumococcal vaccines.

## Introduction

Pneumococcal disease, caused by *Streptococcus pneumoniae*, includes common, noninvasive infections such as otitis media, and severe, life-threatening infections including pneumonia, sepsis and meningitis Pneumococcal disease causes hundreds of thousands of deaths per year worldwide, most of these occurring in children, older adults, and people with immunocompromising conditions. The burden of pneumococcal disease is greatest in low- and middle- income countries, but there are high burdens of disease in infants and older adults in both high- and low-income settings ^1, 2^.

Pneumococcal conjugate vaccines (PCVs) have been shown to have high efficacy against disease and mortality ^3^. Vaccines targeting different serotypes and used with various dosing schedules have been employed in a wide array of populations globally ^4, 5^. With several new pneumococcal vaccines recently licensed and in development, there is a need to compare PCVs and ensure maintenance of vaccine effectiveness ^6– 9^. New PCVs are typically licensed based on comparisons of immunogenicity with existing vaccines. Therefore, there is a need to be able to readily compare the immunogenicity of different PCVs across studies. These immunogenicity data are used by regulators and by national immunization technical advisory groups (NITAGs) to determine which vaccine products, dosing schedules, and age groups will be included in immunization policy recommendations for their respective populations.

Immunogenicity data for pneumococcal vaccine trials should be compared and interpreted with caution, and the web interface has prominent statements about the caveats of the data. Data on immunogenicity alone cannot be used to infer differences in effectiveness between vaccines. These data need to be combined with information on the protective concentration of antibodies required to protect against each serotype in different populations for meaningful comparisons ^10^. Despite these cautions, pulling immunogenicity data together into a single location will make this necessary process of comparison easier and less labor-intensive.

Review and synthesis of the data to inform these policy decisions can be labor intensive and cumbersome. Streamlining this process and making immunogenicity data available in a format that can be readily searched and extracted has value for policy makers and for researchers interested in vaccine efficacy, effectiveness, impact, and optimization. WISSPAR is a resource that provides an interactive platform to summarize and compare the immunogenicity data from clinical trials of pneumococcal vaccines.

## Methods

### Data sources

Data currently displayed on WISSPAR are from the Outcome Measures subsection of the Results section on clinicaltrials.gov. Several other datasets and types are available, including adverse events reporting, subject demographics, retention patterns. There are currently data from more than 57 trials available to compare, though these are updated frequently. If users need data from a trial that is not yet included on WISSPAR, users are encouraged to contact the authors with requests.

### Outcome data

The current version of WISSPAR is focused on two measures of serotype-specific immunogenicity: geometric mean concentrations of immunoglobulin G levels measured by enzyme-linked immunosorbent assay (ELISA) or Electrochemiluminescence (ECL) assay and geometric mean titers measured with opsonophagocytic assays (OPA). These two measurements are commonly taken from blood serum of clinical trial participants at specified time intervals following doses of pneumococcal vaccines (e.g. 1 month following a dose received between 11–15 months of age), and provide a largely standardized basis for comparison, though differences between assays can cause variation. These data are extracted from outcomes tables from clinical trials, and can be filtered, visualized, or exported according to the specifications of the user, including selecting trials of certain vaccine products, manufacturers, dosing schedules, geographic regions, immunocompromised populations, or age groups.

### Dataset validation

Data are manually validated for completeness, clarity and formatting from the customized WISSPAR backend. Data from clinical trials are imported exactly as they are recorded in clinicaltrials.gov, so if data are incorrectly entered into clinicaltrials.gov, the accuracy of WISSPAR could be affected.

### Dashboard contents

**Figure 1.**
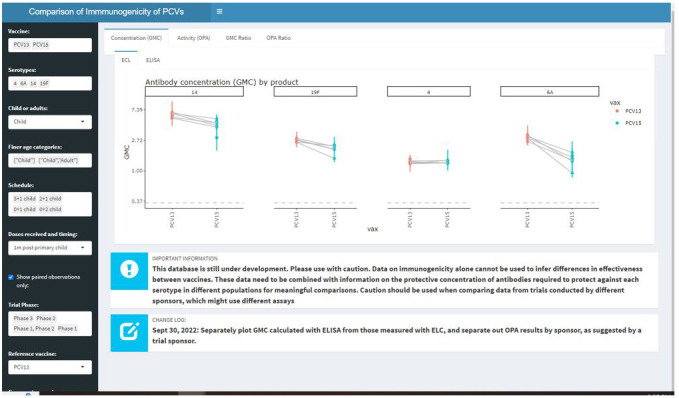
Data visualization app on the Worldwide Index of Serotype-Specific Antibody Responses, WISSPAR. WISSPAR’s data visualization app is based in R Shiny and allows users to generate customizable, downloadable figures showing immunogenicity measurements stratified by serotype, vaccine product, vaccine manufacturer, trial sponsor, and vaccination schedule.

Public-facing WISSPAR content is organized into Data Dashboards, Analysis Tools, and Resources ( Table 1). The Data Dashboards provide customizable visualizations of the serotype-specific immunogenicity data in the Graphical Overview ( Figure 1), and summary information from the included trials in the Clinical Trials Overview. The analysis tools allow users to efficiently pinpoint trials of interest in the Look up a trial tool, and export custom .csv files for downstream analyses using the Immunogenicity Data Export tool. There are community resources available, including a blog to keep users abreast of updates and demonstrate WISSPAR capabilities example analyses. An example of how WISSPAR can be used to make comparisons of serotype-specific differences in immunogenicity between three vaccine products is published on the WISSPAR blog ^11^.

**Table 1.** Descriptions of page content for the Worldwide Index of Serotype-Specific Antibody Responses, WISSPAR. WISSPAR has a suite of tools to summarize, compare, and export immunogenicity data from clinical trials of pneumococcal vaccines.

WISSPAR Website Pages	Content Summary	
**PUBLIC DATA DASHBOARDS**		
**Graphical View**	An interactive Shiny app that allows users to visualize measurements of serotype-specific immunogenicity and export images. Available variables include: • Vaccine • Serotypes • Age category • Schedule • Doses received and timing • Trial phase • Sponsor Users can visualize measurements of IgG Geometric Mean Concentrations (GMCs), Opsonophagocytic Activity assays (OPAs), and GMC and OPA ratios.	
**Clinical Trials Overview**	Filterable listing of clinical trials included on WISSPAR, allowing users to search for particular clinical trials or criteria of interest, including: • Vaccines • Schedule • Manufacturers • Age Category • Ethnicity • Continent • Immunocompromised Groups • Gender	
**ANALYSIS TOOLS**		
**Look up a trial**	Quick-search function that allows users to access complete trial information by entering the NCT ID from clinicaltrials.gov	
**Immunogenicity Data Export**	Highly customizable data export tool where users can create customized datasets for downstream analyses. Variables include:	
	**Filters**	**Fields**
	• Income group • Trial phase • Vaccine • Age category • Immunocompromised group • Responsible party	• Clinical trial data • Study eligibility • Study location • Outcome Measures • Outcome Overview
**RESOURCES**		
**Blog**	Link to WISSPAR blog posts, which includes updates from the team, examples of how to use WISSPAR to compare trial data, and more.	
**Videos**	Video walk-through of WISSPAR features and functions	

### Data hosting and database structure

**Figure 2.**
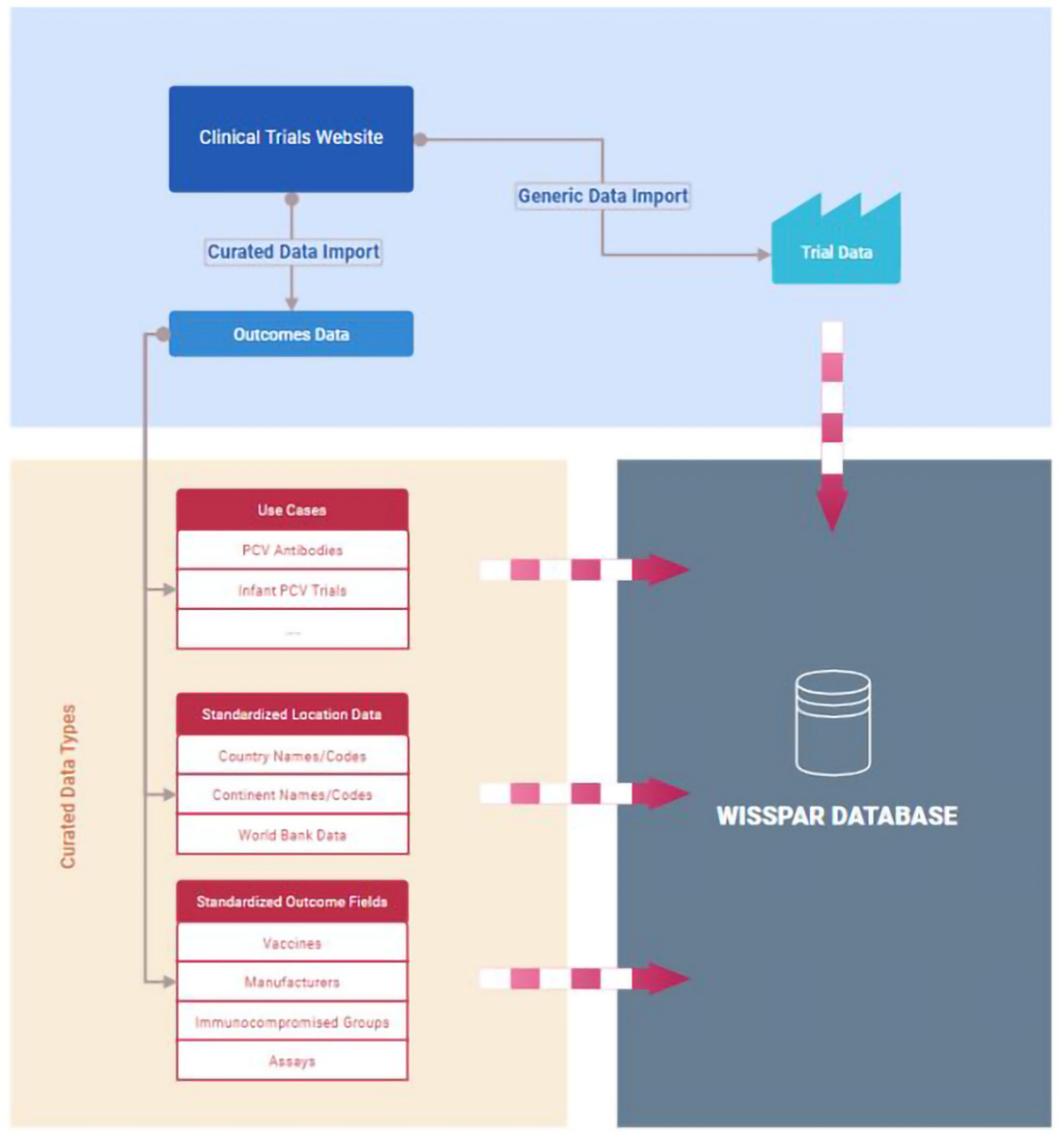
Organizational Schematic of the Worldwide Index of Serotype-Specific Antibody Responses, WISSPAR. The data import process from clinicaltrials.gov and the sources of data and the data types used in WISSPAR are described here.

The WISSPAR platform consists of a few different services hosted on Digital Ocean ( Figure 2). The backend database is PostgreSQL (V13) with connection pools set up for security and guaranteed availability; there are also automated backups for recovery. The web application is written in Golang (1.16) for the backend connecting to the database and integrating with the clinicaltrials.gov website. On the front end of the web application, the javascript framework is SvelteJS to allow for quick component loading times and better code organization over traditional javascript. We used GitLab for our software version control, when a change is made in the repository on GitLab it is detected by our Digital Ocean repository and automatically deployed. We also have fail safes built in so that if a code build fails to deploy it will revert to the last working version without any downtime to the web application. Interactive plots, with filters and selectors, was created in RShiny and deployed through the shiny.io server.

### Use of data

All data on WISSPAR are publicly available or previously published.

## Data Availability

Zenodo: weinbergerlab/WISSPAR: v1_1_1, https://doi.org/10.5281/zenodo.7055186 ^12^. Data are available under the terms of the Creative Commons Attribution 4.0 International license (CC-BY 4.0).
