# Supplementary material for: Worldwide Index of Serotype-Specific Pneumococcal Antibody Responses (WISSPAR): A curated database of clinical trial data
Source: Gates Open Res. 2023 Jul 13;7:109. [Version 1] doi: 10.12688/gatesopenres.14768.1 (PMC10409981; doi:10.12688/gatesopenres.14768.1)
Supplement: Peer review history

# Peer review: Reviewer response for version 1

**DOI:** 10.21956/gatesopenres.16094.r34343

**Published:** 2023-08-15

**Authors:** Fikri Y Avci

**Affiliations:** 1 Emory Vaccine Center, Emory University, Atlanta, Georgia, USA; 2 School of Medicine Department of Biochemistry, Emory University, Atlanta, Georgia, USA

WISSPAR is a much-needed platform that allows quick, comprehensive, and guided access to antibody titers and functional efficacy (OPA) data from worldwide pneumococcal vaccine immunization clinical trials. 

 The authors cite a WISSPAR blog for an example of how WISSPAR can be used and list in Table 1 a video walk-through of WISSPAR, which are highly useful to enable its widespread use. It may be beneficial to add here a step-by-step tutorial for the cited example in a simple protocol format (eg Nat Protoc). Also, in the platform, displaying premade graphs for two or three major clinical trials may give novice users a seed to modulate themselves. CDC's IPD epidemiology platform and WISSPAR complement and appeal to similar users. To increase WISSPAR's visibility, CDC, WHO, and similar platforms can cite it. Finally, allowing user interphase may improve the platform and curate the clinical data. Overall, WISSPAR is a well-implemented important resource.

Are sufficient details of methods and materials provided to allow replication by others?

Yes

Is the rationale for creating the dataset(s) clearly described?

Yes

Are the datasets clearly presented in a useable and accessible format?

Yes

Are the protocols appropriate and is the work technically sound?

Yes

Reviewer Expertise:

Immunology, Infectious Diseases, Glycobiology, Vaccine Design and Development

I confirm that I have read this submission and believe that I have an appropriate level of expertise to confirm that it is of an acceptable scientific standard.

# Peer review: Reviewer response for version 1

**DOI:** 10.21956/gatesopenres.16094.r34280

**Published:** 2023-08-08

**Authors:** Carmen Muñoz-Almagro

**Affiliations:** 1 CIBER of Epidemiology and Public Health (CIBERESP), Madrid, Spain; 2 RDI Microbiology Department, Institut de Recerca Sant Joan de Déu, Hospital Sant Joan de Déu, Barcelona, Catalonia, Spain; 3 Medicine Department, Universitat Internacional de Catalunya, Barcelona, Catalonia, Spain

The Worldwide Index of Serotype Specific Pneumococcal Antibody Responses (WISSPAR) is a helpful and comprehensive online tool including immunogenicity data from clinical trials of pneumococcal vaccines (PCVs) according to numerous vaccine products, manufacturers, dosing schedules, age groups, immunocompromised groups, and geographic regions.

 The platform is user-friendly and without a doubt will be a very useful tool for researchers, regulators and technical advisory groups that need to compare the immunogenicity of different PCVs across studies. At present data of WISSPAR come from more than 57 trials but  WISSPAR curators update the database frequently. In addition, if users need data from a trial that is not yet included on WISSPAR, they are encouraged to contact the authors with requests. Measures of serotype-specific immunogenicity include IgG Geometric Mean Concentrations (GMCs), Opsonophagocytic Activity assays (OPAs), and GMC and OPA ratio.

 My only minor concern is about data validation: data from clinical trials are imported exactly as they are recorded in clinicaltrials.gov, so if data are incorrectly entered into clinicaltrials.gov, the accuracy of WISSPAR could be affected. My suggestion would be including an alert if they or the scientific community detect any data that might reasonably considered to be erroneous. On the other hand, it could be interesting to provide a brief audit on the methodological quality of data reported in the clinical trials included in the tool, using widely accepted review checklists such as those of CONSORT reporting guidelines.

Are sufficient details of methods and materials provided to allow replication by others?

Yes

Is the rationale for creating the dataset(s) clearly described?

Yes

Are the datasets clearly presented in a useable and accessible format?

Yes

Are the protocols appropriate and is the work technically sound?

Yes

Reviewer Expertise:

Pediatric Infectious Diseases; molecular epidemiology;  invasive pneumococcal disease

I confirm that I have read this submission and believe that I have an appropriate level of expertise to confirm that it is of an acceptable scientific standard.

# Peer review: Reviewer response for version 1

**DOI:** 10.21956/gatesopenres.16094.r34277

**Published:** 2023-08-02

**Authors:** Giuseppe Ercoli

**Affiliations:** 1 Centre for Inflammation and Tissue Repair, UCL Respiratory, University College London, London, UK

The WISSPAR database presented by Perniciaro *et al.,* provides an extremely valuable tool to keep track of clinical trials data on pneumococcal vaccines. The tool allows any user to easily access the clinical trial data and compare different datasets. Having a tool that allows to compare PCVs and assess vaccine effectiveness would greatly help vaccine research. The new resource is especially useful for vaccines in early development stages or discovery phase, providing access and facilitating the comparison to previously published data. The rationale for creating the dataset is clearly described by the authors and both the methodology and data sources are reported in details.

 Overall the database presented is certainly suitable for indexing, my only comment would be about future updates. It would be interesting to know if the authors have already planned how future updates of the system will be released. If new datasets will become available, will they be immediately included? What's the planned frequency of the updates? Will the frequency of the system updates be time-dependent or data-dependent? Is there any plan to add any extra function?

Are sufficient details of methods and materials provided to allow replication by others?

Yes

Is the rationale for creating the dataset(s) clearly described?

Yes

Are the datasets clearly presented in a useable and accessible format?

Yes

Are the protocols appropriate and is the work technically sound?

Yes

Reviewer Expertise:

Vaccine development / infectious diseases

I confirm that I have read this submission and believe that I have an appropriate level of expertise to confirm that it is of an acceptable scientific standard.
